# Supplementary material for: The Systems Biology Research Tool: evolvable open-source software
Source: BMC Syst Biol. 2008 Jun 29;2:55. doi: 10.1186/1752-0509-2-55 (PMC2446383; doi:10.1186/1752-0509-2-55)
Supplement: Additional file 1 — SBRT Archive. An archive of the current version of the Systems Biology Research Tool. [file 1752-0509-2-55-S1.zip › sbrt-1.4.0/doc/developers_guide/api/sbrt/shell/text/class-use/Parser.html]

Uses of Interface sbrt.shell.text.Parser


|  |  |  |
| --- | --- | --- |
| **Overview** \| **Package** \| **Class** \| **Use** \| **Tree** \| **Deprecated** \| **Index** \| **Help** | |  |
| PREV   NEXT | **FRAMES**    **NO FRAMES**     **All Classes** |  |


---


## **Uses of Interface sbrt.shell.text.Parser**

| Packages that use Parser | |
| --- | --- |
| **sbrt.shell.io** | Provides classes and interfaces for reading and writing files. |
| **sbrt.shell.text** | Provides classes and interfaces for defining formats for basic objects. |
| **sbrt.shell.text.chem** | Provides classes and interfaces for defining formats for chemistry related objects. |
| **sbrt.shell.text.fba** | Provides classes and interfaces for defining formats for objects relevant to Flux Balance Analysis. |
| **sbrt.shell.text.math** | Provides classes and interfaces for defining formats for mathematical objects. |

Uses of Parser in sbrt.shell.io

| Methods in sbrt.shell.io that return Parser | |
| --- | --- |
| `Parser<E>` | `TextInputFile.getLineFormat()`             Returns the format used to parse lines of this file. |

Constructors in sbrt.shell.io with parameters of type Parser

`TextInputFile(java.lang.String fileName,
FileFormat fileFormat,
Parser<E> fileLineFormat)`
  
          Constructs a new text input file using the provided arguments.

`TextInputFile(java.lang.String fileName,
FileFormat fileFormat,
Parser<E> fileLineFormat,
java.util.Collection<java.lang.Character> commentCharacters)`
  
          Constructs a new text input file using the provided arguments.

`TextInputFile(java.lang.String fileName,
Parser<E> fileLineFormat)`
  
          Constructs a new text input file using the provided arguments.

Uses of Parser in sbrt.shell.text

| Subinterfaces of Parser in sbrt.shell.text | |
| --- | --- |
| `interface` | `BooleanFormat`             This interface is used to represent a format for boolean values. |
| `interface` | `CollectionFormat<E>`             This interface is used to represent collection formats. |
| `interface` | `DoubleFormat`             This interface is used to represent a format for double precision numbers. |
| `interface` | `IntegerFormat`             This interface is used to represent a format for integers. |
| `interface` | `LinkedHashSetFormat<E>`             This interface is used to represent `LinkedHashSet` formats. |
| `interface` | `ListFormat<E>`             This interface is used to represent list formats. |
| `interface` | `LongFormat`             This interface is used to represent a format for longs. |
| `interface` | `PercentFormat`             This interface is used to represent a format for percentages. |
| `interface` | `SimpleFormat<T>`             This interface is used to represent simple formats. |

| Classes in sbrt.shell.text that implement Parser | |
| --- | --- |
| `class` | `AbstractLinkedHashSetFormat<E>`             This abstract class is a skeleton implementation of `LinkedHashSetFormat`. |
| `class` | `AbstractListFormat<E>`             This abstract class is a skeleton implementation of `ListFormat`. |
| `class` | `AmpersandSetFormat<E>`             This class is used to define a format for ampersand-delimited sets. |
| `class` | `BoolRelFormatV1<E>`             This interface is used to represent boolean relationship formats. |
| `class` | `DoubleFormatV1`             This class is a concrete implementation of `DoubleFormat`. |
| `class` | `DoubleFormatV2`             This class is a concrete implementation of `DoubleFormat`. |
| `class` | `FileFormatFormat`             This class is used to represent formats for types of file formats. |
| `class` | `IntegerFormatV1`             This class is a concrete implementation of `IntegerFormat`. |
| `class` | `LongFormatV1`             This class is a concrete implementation of `LongFormat`. |
| `class` | `NonEmptyStringFormat`             This class is used to define a format for `String` objects. |
| `class` | `PercentFormatVersion1`             This class is a concrete implementation of `PercentFormat`. |
| `class` | `PipeListFormat<E>`             This class is used to define a format for pipe-delimited lists. |
| `class` | `PipeSetFormat<E>`             This class is used to define a format for pipe-delimited sets. |
| `class` | `SimpleListFormat<E>`             This interface is used to wrap `ListFormat` objects as `SimpleFormat` objects. |
| `class` | `SimpleSetFormat<E>`             This interface is used to wrap `ListFormat` objects as `SimpleFormat` objects. |
| `class` | `StringFormat`             This class is used to define a format for `String` objects. |
| `class` | `TabListFormat<E>`             This class is used to define a format for tab-delimited lists. |
| `class` | `TabSetFormat<E>`             This class is used to define a format for tab-delimited sets. |
| `class` | `TrueFalseFormat`             This class is used to define a format for true/false values. |
| `class` | `WhitespaceListFormat<E>`             This class is used to define a format for whitespace-delimited lists. |
| `class` | `WhitespaceSetFormat<E>`             This class is used to define a format for whitespace-delimited sets. |
| `class` | `YesNoFormat`             This class is used to define a format for yes/no values. |

| Methods in sbrt.shell.text that return Parser | |
| --- | --- |
| `Parser<E>` | `ListFormat.getElementParser()`             Returns the parser for the elements of this list format. |
| `Parser<E>` | `AbstractListFormat.getElementParser()`             Returns the parser for the elements of this set format. |
| `Parser<E>` | `BoolRelFormatV1.getElementParser()`             Returns the parser for the elements of this set format. |
| `Parser<E>` | `SimpleSetFormat.getElementParser()`             Returns the parser for the elements of this set format. |
| `Parser<E>` | `SimpleListFormat.getElementParser()`             Returns the parser for the elements of this list format. |
| `Parser<E>` | `AbstractLinkedHashSetFormat.getElementParser()`             Returns the parser for the elements of this set format. |
| `Parser<E>` | `LinkedHashSetFormat.getElementParser()`             Returns the parser for the elements of this set format. |
| `Parser<K>` | `AbstractMapFormat.getKeyParser()`             Returns the parser used for keys. |
| `Parser<V>` | `AbstractMapFormat.getValueParser()`             Returns the parser used for values. |

Constructors in sbrt.shell.text with parameters of type Parser

`AbstractLinkedHashSetFormat(java.lang.String delimiter,
java.util.regex.Pattern regex,
Formatter<E> elementFormatter,
Parser<E> elementParser)`
  
          Constructs a new `LinkedHashSet` format from
the provided arguments.

`AbstractListFormat(java.lang.String delimiter,
java.util.regex.Pattern regex,
Formatter<E> elementFormatter,
Parser<E> elementParser)`
  
          Constructs a new list format from the provided arguments.

`AmpersandSetFormat(Formatter<E> elementFormatter,
Parser<E> elementParser)`
  
          Constructs a new ampersand-delimited set format with the
provided element format.

`EqualsMapFormatV1(Formatter<K> keyFormatter,
Parser<K> keyParser,
Formatter<V> valueFormatter,
Parser<V> valueParser)`
  
          Constructs a new map format using the provided key and
value formats.

`EqualsMapFormatV1(Formatter<K> keyFormatter,
Parser<K> keyParser,
Formatter<V> valueFormatter,
Parser<V> valueParser)`
  
          Constructs a new map format using the provided key and
value formats.

`PipeListFormat(Formatter<E> elementFormatter,
Parser<E> elementParser)`
  
          Constructs a new pipe-delimited list format with the
provided element format.

`PipeSetFormat(Formatter<E> elementFormatter,
Parser<E> elementParser)`
  
          Constructs a new pipe-delimited set format with the
provided element format.

`TabListFormat(Formatter<E> elementFormatter,
Parser<E> elementParser)`
  
          Constructs a new tab-delimited list format with the
provided element format.

`TabSetFormat(Formatter<E> elementFormatter,
Parser<E> elementParser)`
  
          Constructs a new tab-delimited set format with the
provided element format.

`WhitespaceListFormat(Formatter<E> elementFormatter,
Parser<E> elementParser)`
  
          Constructs a new whitespace-delimited list format with the
provided element format.

`WhitespaceSetFormat(Formatter<E> elementFormatter,
Parser<E> elementParser)`
  
          Constructs a new whitespace-delimited set format with the
provided element format.

Uses of Parser in sbrt.shell.text.chem

| Subinterfaces of Parser in sbrt.shell.text.chem | |
| --- | --- |
| `interface` | `CatalystFormat`             This interface is used to represent the formats of catalysts in chemical reactions. |
| `interface` | `ChemSpeciesFormat`             This interface is used to represent the formats of chemical species. |
| `interface` | `RxnNameFormat<T>`             This interface is used to represent formats for the names of chemical reactions. |

| Classes in sbrt.shell.text.chem that implement Parser | |
| --- | --- |
| `class` | `CatalystFormatV1`             This class is a concrete implementation of `CatalystFormat`. |
| `class` | `CatalystFormatV2`             This class is a concrete implementation of `CatalystFormat`. |
| `class` | `ChemSpeciesFormatV1`             This class is a concrete implemenation of `ChemSpeciesFormat`. |
| `class` | `IrrevRxnFormatV1`             This class is used to format irreversible chemical reactions. |
| `class` | `IrrevRxnFormatV2`             This class is used to format irreversible chemical reactions. |
| `class` | `RevRxnFormatV1`             This class is used to format reversible chemical reactions. |
| `class` | `RevRxnFormatV2`             This class is used to format reversible chemical reactions. |
| `class` | `RxnNameFormatV1`             This class is a concrete implementation of `RxnNameFormat`. |

Uses of Parser in sbrt.shell.text.fba

| Subinterfaces of Parser in sbrt.shell.text.fba | |
| --- | --- |
| `interface` | `CatalystListFormat`             This interface is used to represent the formats of lists of catalyst names. |
| `interface` | `CatalystSetFormat`             This interface is used to represent the formats of sets of enzyme names. |
| `interface` | `FbaOptHeaderParser`             This interface is used to represent parsers for headers used in writing files of FBA optimization data. |
| `interface` | `RxnNameExprFormat<E extends MathExpr<S>,S>`             This interface is used to represent formats for mathematical expressions of reaction names. |
| `interface` | `RxnNameListFormat`             This interface is used to represent the formats of collections of reaction names. |
| `interface` | `RxnNameOrExprFormat`             This interface is used to represent formats for reaction names and mathematical expressions of reaction names. |
| `interface` | `RxnNameSetFormat`             This interface is used to represent the formats of collections of reaction names. |

| Classes in sbrt.shell.text.fba that implement Parser | |
| --- | --- |
| `class` | `CatalystListFormatV1`             This class is a concrete implemenation of `CatalystListFormat`. |
| `class` | `CatalystSetFormatV1`             This class is a concrete implemenation of `CatalystSetFormat`. |
| `class` | `CatalystVerifier`             This class is used to ensure catalysts exist in a given `CatalyzedFluxome`. |
| `class` | `ChemSpeciesVerifier`             This class is used to ensure chemical species exist in a given `Fluxome`. |
| `class` | `FbaOptHeaderParserV1`             This class is a concrete implemenation of `FbaOptHeaderParser`. |
| `class` | `RxnNameExprFormatV1`             This class is used to format linear combinations of reaction names. |
| `class` | `RxnNameListFormatV1`             This class is a concrete implemenation of `RxnNameListFormat`. |
| `class` | `RxnNameOrExprFormatV1`             This class is a concrete implemenation of `RxnNameOrExprFormat`. |
| `class` | `RxnNameSetFormatV1`             This class is a concrete implemenation of `RxnNameSetFormat`. |
| `class` | `RxnNameVerifier`             This class is used to ensure reaction names exist in a given `Fluxome`. |

Uses of Parser in sbrt.shell.text.math

| Subinterfaces of Parser in sbrt.shell.text.math | |
| --- | --- |
| `interface` | `CoeffFormat`             This interface is used to represent the format of coefficients of mathematical expressions. |
| `interface` | `DirEdgeFormat<N>`             This interface is used to represent formats for the edges of directed graphs. |
| `interface` | `IndexPairFormat<R,C>`             This interface is used to represent formats for pairs of indices. |
| `interface` | `IntervalFormat`             This interface is used to represent formats for closed intervals. |
| `interface` | `LinearCombFormat<V>`             This interface is used to represent formats for linear combinations. |
| `interface` | `LinearCombTermFormat<V>`             This interface is used to represent the formats of terms from linear combinations. |
| `interface` | `MathExprFormat<E extends MathExpr<V>,V>`             This interface is used to represent formats for linear combinations. |
| `interface` | `MatrixElementFormat<R,C,E>`             This interface is used to represent formats for matrix elements. |

| Classes in sbrt.shell.text.math that implement Parser | |
| --- | --- |
| `class` | `BoundedIntervalFormatV1`             This class is a concrete implementation of `IntervalFormat`. |
| `class` | `CoeffFormatV1`             This class is a concrete implementation of `CoeffFormat`. |
| `class` | `CoeffFormatV2`             This class is a concrete implementation of `CoeffFormat`. |
| `class` | `DirEdgeFormatV1<N>`             This class is a concrete implementation of `DirEdgeFormat`. |
| `class` | `IndexPairFormatV1<R,C>`             This class is a concrete implementation of `IndexPairFormat`. |
| `class` | `IntervalFormatV1`             This class is a concrete implementation of `IntervalFormat`. |
| `class` | `LinCombTermFormatV1<V>`             This class is a concrete implementation of `LinearCombTermFormat`. |
| `class` | `LinCombTermFormatV2<V>`             This class is a concrete implementation of `LinearCombTermFormat`. |
| `class` | `LinearCombFormatV1<V>`             This class is a concrete implementation of `LinearCombFormat`. |
| `class` | `LinearCombFormatV2<V>`             This class is a concrete implementation of `LinearCombFormat`. |
| `class` | `MatrixElementFormatV1<R,C>`             This class is a concrete implementation of `MatrixElementFormat`. |
| `class` | `SenseFormat`             This class is used to represent formats of optimization senses. |

---


|  |  |  |
| --- | --- | --- |
| **Overview** \| **Package** \| **Class** \| **Use** \| **Tree** \| **Deprecated** \| **Index** \| **Help** | |  |
| PREV   NEXT | **FRAMES**    **NO FRAMES**     **All Classes** |  |


---
